# Successful treatment with tocilizumab every 4 weeks of a low disease activity group who achieve a drug-free remission in patients with systemic-onset juvenile idiopathic arthritis

**DOI:** 10.1186/1546-0096-13-4

**Published:** 2015-01-23

**Authors:** Mikhail M Kostik, Margarita F Dubko, Vera V Masalova, Ludmila S Snegireva, Tatyana L Kornishina, Irina A Chikova, Eugenia A Isupova, Ekaterina M Kuchinskaya, Natalia I Glebova, Natalia V Buchinskaya, Olga V Kalashnikova, Vyacheslav G Chasnyk

**Affiliations:** Hospital Pediatric Department, Saint-Petersburg State Pediatric Medical University, Lytovskaya 2, Saint-Petersburg, 194100 Russia

**Keywords:** Systemic-onset juvenile idiopathic arthritis, Interleukine-6, Tocilizumab, Biologic free remission, Low disease activity, High disease activity

## Abstract

**Background:**

Systemic juvenile idiopathic arthritis (SoJIA) is the most striking form of juvenile idiopathic arthritis. The aim of our study was to evaluate the clinical responses and outcomes of children with SoJIA to IL-6 blockade using two different tocilizumab (TCZ) treatment protocols designed for milder and more severe SoJIA patient groups, and evaluate the possibility of achieving biologic-free remission.

**Methods:**

Thirty-seven active SoJIA children who have failed treatment with corticosteroids and other DMARDs were included in our retrospective study. TCZ doses were prescribed in two treatment approaches: every 2 weeks TCZ dosing (Q2W) and every 4 weeks TCZ dosing (Q4W). The patients were assigned to these two groups by the study physicians depending on the severity of the SoJIA disease as judged by each clinician.

**Results:**

Thirty-three of the 37 children successfully completed the trial. TCZ was discontinued in 11patients during the trial. Seven children achieved inactive disease and were allowed to stop the TCZ and 4 had severe adverse events requiring drug cessation. Currently 7 patients continue to have TCZ-free remission [4/7 remission off-medication, 3/7still on methotrexate (MTX)]. This mixed group had a median treatment duration of 1002 days. The children in remission off of all medications, TCZ and MTX, had a median remission duration of 1162 days (ranged 932–1301 days).

Compared to the patients assigned to the Q2W TCZ treatment group, the patients assigned to the Q4W TCZ group had a milder SoJIA course. The patients had higher levels of hemoglobin, total proteins, and serum albumins. They had lower white blood cell counts (WBC), % granulocytes, CRP, ESR, ferritins, and LDH. These children had a lower frequency of internal organ involvement, fewer relapses during TCZ treatment, and no macrophage activation syndrome episodes.

**Conclusions:**

Our experience with TCZ for SoJIA supports the excellent result of other studies. What may be novel is our finding that thisIL-6 blockade with TCZ may be able to be utilized at a less frequent dosing schedule in mild SoJIA compared to severe SoJIA. We discuss other factors that may increase the probability of a patient reaching TCZ-free remission.

## Background

Systemic-onset juvenile idiopathic arthritis (SoJIA) is the most striking forms of juvenile idiopathic arthritis. This challenging disease unchecked may lead to severe joint disability and internal organ involvement and is frequently associated with life-threatening complications such as macrophage activation syndrome and amyloidosis [1]. There are typical SoJIA-related long-term adverse events that have been noted for decades, both from the disease and the treatment with corticosteroids. These include anemia, Cushing’s syndrome, obesity, growth failure, osteoporosis with pathological fractures, aseptic bone necrosis, hypertension as well as metabolic disturbances such as hyperglycemia and dyslipidemia [2]. Due to the failure of corticosteroids (CS) and DMARDs such as MTX to adequately control SoJIA and difficult side effects of these medications in many children, rheumatologists have recently begun treating SoJIA patients with biologic medications despite the high cost of the drugs [3–7].

Biologic medications that provide blockade of interleukin-1 (Il-1) and interleukin-6 (Il-6) appear to be most effective current treatment of children with SoJIA in 2014. They provide impressive control of SoJIA disease activity in approximately 2/3’s of patients with SoJIA [3–6]. The increasing use of these biologics had led to a dramatic improvement in the short-term outcome of SoJIA patients [4–7]. Unfortunately, Il- 1and IL-6 blockers in many countries are still not available and/or affordable. In our country, the IL-6 blocker is the only biologic drug available for SoJIA management at this time.

Recent studies of the pathophysiology of SoJIA have shown an important role for Il-6 in joint inflammation. IL-6 also appears to have a major factor in systemic features, such as rash, serositis, lymphadenopathy, and hepatosplemomegaly [8–10]. Two major drug trials performed initially by S.Yokota and co-workers in Japan and later in USA and Europe (TENDER trial) have supported the efficacy of IL-6 blockade in SoJIA [4–6]. Since IL-1 blockers were unavailable in Russia at the time of this study and tocilizumab (TCZ) was registered and approved for adults with rheumatoid arthritis (RA), we have been able to use TCZ off label for treatment of SoJIA, as the only option for management of SoJIA in patients unresponsive to other medications.

When we begun to use TCZ at our center, the data about how frequently to administer TCZ for children with SoJIA was limited. There was only data of S. Yokota’s study, a study with a single TCZ infusion only, and studies of TCZ administration in rheumatoid arthritis (RA) in adults [4, 5, 11, 12]. In the Japanese study, TCZ was administered every 2 weeks, while in adults with RA TCZ was used every 4 weeks. P. Woo and co-workers showed the 8-week efficacy of single 8 mg/kg dose of TCZ. Later in the CHERISH study and in a Japanese study about TCZ in polyarticular-course JIA, TCZ was given every 4 weeks [13, 14].

The aim of our study was to review our use of TCZ at our clinic and evaluate the children’s clinical response to IL-6 blockade. We used 2 TCZ treatment protocols and evaluated outcomes including the optimal possibility of achieving biologic-free remission.

## Methods

### Study design and patient selection

In our retrospective study, we reviewed the medical records of 37 children with active SOJIA. Diagnosis of SoJIA was based on ILAR definitions [15]. TCZ was initiated in each child at a dosage of 12 mg/kg if the child’s weight was < 30 kg and 8 mg/kg if the weight was ≥30 kg. Active disease was defined if patients had any clinical or laboratory abnormalities attributed to SoJIA. These features included synovitis, an intermittently spiking fever, rash, polyserositis, lymphadenopathy, hepatosplenomegaly, a C-reactive protein (CRP) level >15 mg/l, and an erythrocyte sedimentation rate (ESR) > 30 mm/hour.

The main inclusion criteria were:

1. An inadequate response or intolerance of DMARDs and their combination;
2. Inability to sufficiently taper corticosteroids due to flares of systemic features or synovitis or both;
3. The presence of corticosteroid-related side effects.

For assessment of efficacy we used the six variables of the ACR core set for JIA: the number of joints with active arthritis, the number of joints with limited range of motion, the physician’s global assessment of disease activity, the parent’s global assessment of overall well-being and physical function utilizing the Disability Index of the Childhood Health Assessment Questionnaire, and the erythrocyte sedimentation rate [16]. Fever was assessed by measurement of the axillar temperature at least two times a day. Only patients who achieved at least ACRPedi30 response at day 14 could continue the study (improvement of 30% or more in three or more of the six variables of the ACR core set for JIA, with no more than 1 variable worsening by more than 30% and absence of the fever). Similarly, the ACRPedi70 response is defined as improvement of at least 70% in at least three of the six core criteria for JIA, with worsening of more than 30% in no more than one criterion.

We used TCZ in 2 dose schedules: every 2 weeks (Q2W) and every 4 weeks (Q4W). Each clinician made the dose schedule decision based upon the severity of disease, steroid-dependence, presence of MAS and organ involvement. All patients that developed MAS immediately precede TCZ were treated only with Q2W TCZ. Initially 29 patients were treated Q4W and 3 of them were transferred in the Q2W group due to clinical worsening, defined as a clinical relapse or exacerbation after the initial improvement). Only patients who had no signs of flare, exacerbation, side effects or TCZ inefficacy during Q4 week treatment could continue to receive TCZ Q4W (n = 26).

**Figure 1:**
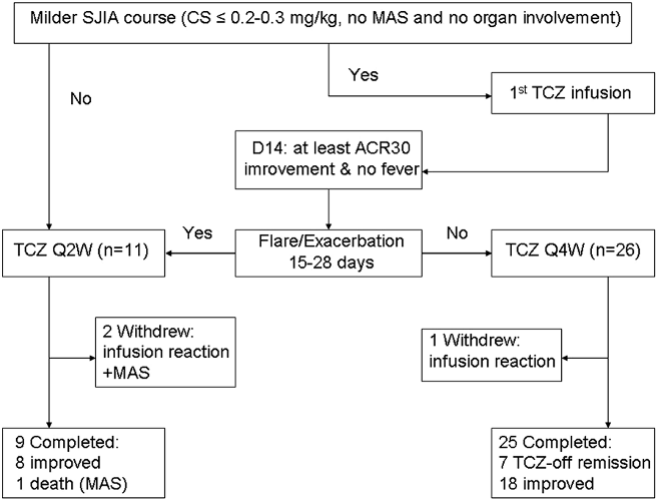
**Flow chart of the study.**

Eight SoJIA patients with severe disease were initially treated with Q2W TCZ. The number increased to eleven patients who were considered to have severe SoJIA with the addition of 3 children from the TCZ Q4W group during the first 4 weeks or later of TCZ treatment. The duration of study was from the times of the 1st and last TCZ infusions of each patient.The flow chart of our study is shown in Figure 1. The protocol of this trial was approved by local Ethic Committee of our University.

We collected the main SoJIA-related clinical and laboratory data before 1st TCZ infusion. The diagnosis of macrophage activation syndrome (MAS), was based on the Ravelli criteria of 2002 and 2005 [17, 18].

We evaluated all SoJIA-related laboratory data before 1st TCZ infusion including hemoglobin (Hb), white blood cells (WBC), % granulocytes count, platelets (PLT), Westergren ESR, CRP, ferritin, lactate dehydrogenize (LDH) activity, total serum protein, and albumin levels. Additionally, we performed WBC and % granulocytes count testing in 1, 2 and 4 week after the 1st TCZ infusion.

The number of active joints was recorded at the beginning of TCZ treatment. Inactive disease was defined according to the Wallace criteria (2004): no joints with active arthritis, no fever (body temperature ≤38°C), and no rheumatoid rash, Inactive disease also required serositis, splenomegaly, hepatomegaly or generalized lymphadenopathy attributable to JIA, a normal ESR or CRP, and a physician’s global assessment of disease activity indicating no disease activity (i.e. best possible score ≤10 mm*) [19]. In our patients, a TCZ-free remission meant that our patient had at least 12 month course of TCZ, achieved the status of inactive disease, successfully tapered off corticosteroids, stopped TCZ and had at least 6 month period of inactive disease after the last TCZ infusion.

### Statistics

Descriptive statistics were reported in terms of medians (Me) and interquartile ranges (IQRs) for continuous variables and in terms of absolute frequencies and percentages for categorical variables. We utilized Mann–Whitney U-test for comparison of quantitative variables in two groups and chi-square test for comparison of qualitative data, or the Fisher’s exact test in case of expected frequencies < 5. The ability of each variable to discriminate the Q4W from Q2W was evaluated with sensitivity and specificity analysis, AUC-ROC (area under receiver operating characteristic) curve with 95% confidence interval (CI), calculating odds ratio (OR) for detection of the best cut-offs of continuous variables. The higher values of OR of variables interfere better with discriminatory ability. For laboratory tests we used the AUC-ROC analysis with 95% CI. For each categorical variable the analysis of sensitivity and specificity was performed. We used the “best” threshold obtained for the ROC curve analysis of our data because they provide the most appropriate mean between sensitivity and specificity.

Survival analysis with for achievement of the TCZ-free status due to remission as the event of interest was conducted by means of the Kaplan-Meier method. Survival curves were compared by the logrank test. Factors significantly associated with time to achievement TCZ-free status were then tested in a Cox proportional hazards regression model. P < 0.05 was considered as statistically significant. The software Statistica (release 6.0, StatSoft Corporation, Tulsa, OK, USA), Biostat, and MedCalc and were used for data analyses. P-values < 0.05 were considered to indicate a significant difference.

## Results

The main demographic parameters included the median age (Me; IQR) of TCZ start of 10.2 years (range 6.0-12.8) and the median delay from diagnosis to use of TCZ if 36.0 months (range 10.7-97.0). The median duration of TCZ treatment was 665 (range 456–1000) days. Thirty-three of 37 children (89.2%) successfully completed the treatment program. In the beginning of the treatment of 4 patients, one died and 3 withdrew due to serious adverse events and stopped the TCZ. Parameters were calculated in groups that consisted of 37, 36 or 33 patients. Some patients included in the trial had few if any systemic features. Some patients also may have had no active arthritis in the trial. Both milder disease conditions may have been due to disease course or concomitant treatment (e.g., corticosteroids ). Detailed data are in Table 1.

**Table 1:** **Study population**

Parameters	Me (IQR), n = 37
Females, n (%)	21/37 (56.8)
Age of the start TCZ, years	10.2 (6.0-12.8)
TCZ delay, months	36.0 (10.7-97.0)
SoJIA related symptoms at the start of TCZ, n (%)	
- fever	28 (75.7)
- rash	24 (73.0)
- hepatomegaly	20 (54.1)
- lymphadenopathy	13 (35.1)
- splenomegaly	11 (29.7)
- heart involvement	10 (27.0)
- interstitial lung disease	6 (16.2)
- CNS dysfunction	6 (16.2)
- coagulopathy with hemorrhage	4 (10.8)
Hemoglobin, g/dl	11.0 (10.3 – 12.1)
WBC, *10^9^/l	11.9 (7.9 – 16.1)
Granulocytes, cells in 1 μl	7812.0 (5530.0 – 13202.0)
CRP, mg/l	35.0 (11.6 – 88.5)
ESR, mm/h	42.0 (22.0 – 54.0)
Platelets *10^9^/l	361.0 (299.0 – 465.0)
Ferritin, mg/ml	197.0 (84.0 – 841.0)
LDH, U/l	513.0 (425.0 – 743.0)
Albumin, g/dl	3.0 (2.5 – 3.2)
Number of active joints at the start of TCZ	7.0 (2.0 – 22.0)
no active joints, n (%)	4 (10.8)
< 5 active joints, n (%)	13 (35.1)
≥5 active joints, n (%)	20 (54.1)
Treatment before TCZ administration:	
- Corticosteroids, n (%)	26 (70.3)
- mean dose, mg/kg	0,73
- Methotrexate, n (%)	32 (86.5)
- mean dose, mg/m^2^/week	14,7
- Cyclosporine A, n (%)	20 (54.1)
- mean dose, mg/kg	4,2
Combination	18 (48.7)
MAS before TCZ administration, n (%)	12 (32.4)

*Pts # 27, 32 have developed infusion reaction and MAS coincidently; pt # 20 had only infusion reaction leads to TCZ discontinuation, Me – median, IQR –interquartile ranges.

Macrophage activation syndrome (MAS) occurred before TCZ was in 12 children (32.4%). During the trial CS was successfully discontinued in 21/26 (80.8) after a mean of 66.0 days (range 43.0 – 93.0). CsA was discontinued in 10/20 (50.0%) after a mean of 53 days (range 25–85) after achievement of improvement, MTX in 9/32 (28.1%) patients after a mean of 11.5 months (range 3.8 – 25.6). During the trial 11/33 (33.3%) patients accomplished the status of being only on the monotherapy of TCZ.

Tocilizumab was discontinued in 11 patients during the trial. The reasons for stopping TCZ were achievement of inactive diseases status, severe adverse events and death. Inactive disease was reached in 12 patients, and TCZ treatment was stopped in 7/33 (21.2%) children. Severe adverse events (SAE) included infusion reactions and a diagnosis of early MAS. These problems lead to withdrawal of TCZ treatment patients after the 2nd or 3rd infusions; three patients were from the Q2W group and 1 case of infusion reaction was in the Q4W group. Interestingly, 2 of these patients have developed both infusion reaction and MAS at the same time. One patient had an infusion reaction only. All infusion reactions were stereotypical: chill, pallor, acrocyanosis, arterial hypotension. After TCZ initiation, 5 (13.6%) of the children developed MAS. All 5 had the MAS in their past medical history immediately before 1st TCZ infusion, so no new cases developed on TCZ. Two children withdrew (see above). One child (2.7%) died in 5th month of TCZ therapy due to severe uncontrolled MAS. In patients who developed MAS we had used a combination of corticosteroids, IVIG and TCZ treatment.

In children who achieved inactive disease by our evaluation, were successfully weaned off of corticosteroids, CsA and MTX, and had at least a 12 month TCZ course, we tried to initiate a gradual taper of the TCZ. At first we elongated period between infusions. We increased the interval between infusions up to 5 weeks during the next 3–4 months, then did infusions every 6 weeks during the next 3 months. If at that point, the patient had no any clinical and laboratory signs of SoJIA disease activity, TCZ therapy was stopped. In some patients MTX was discontinued before TCZ was stopped but in other patients MTX was continued during TCZ tapering and after the TCZ treatments were discontinued.

Only an attending physician could decide whether to stop the MTX. Usually this decision to continue or stop the MTX was based on the number of active joints at the start of TCZ, the presence of erosions at onset of TCZ treatment and how long the delay was between SoJIA onset and the start of the TCZ. At the end of this study, 7/33 (21.2%) patients were in a TCZ remission (4/7 remission off all medications, 3/7 in remission but still on MTX). The median time in remission for those off of all medications including TCZ was 1078 days (IQR: 848–1217) days. For the children off of TCZ but still on MTX, the median duration 918 days (IQR: 508–1078).

The longest period of time off TCZ and in remission in our cohort was 1467 days. Also we have another 5/12 children who started TCZ later in our study, who have achieved inactive disease, and currently receive TCZ every 5 or 6 weeks. The plans are to stop TCZ in future. Demographics and outcomes are in Table 1.

A comparison of clinical and laboratory features between patients with Q2W and Q4W group are in the Table 2. We have not found any differences between both groups in gender distribution, and frequency of CS, MTX and CsA usage. Also there were no differences in such SoJIA-related signs and symptoms as fever, rash, and lymphadenopathy. As suspected, patients who were treated every 4 weeks had typically a higher Hb, total protein, albumin as well as a lower WBC, % granulocytes, CRP, ESR, ferritin, and LDH.

**Table 2:** **Comparative analysis between SoJIA children which were treated every 2 and every 4 weeks**

Parameters	Q2W (n =11)	Q4W (n =26)	р
Data of baseline			
Hemoglobin, g/dl	9.8 (8.16; 11.6)	11,4 (10,8; 13,0)	0,006
Anemia, n (%)	9 (81.8)	12 (46,2)	0,07*
WBC, *10^9^/l	17.2 (12.8; 20.8)	9,85 (7,8; 13,8)	0,008
WBC in 1 week, *10^9^/l	11.6 (8.2; 19.0)	7.6 (5.2; 11.9)	0.05
Granulocytes, cells in 1 μl	13728 (10112; 18654)	6445 (4914; 8787)	0,002
Granulocytes in 1 week, cells 1 μl	8944 (6560; 15390)	3314 (1840; 7240)	0,005
Granulocytes in 2 weeks, cells 1 μl	8925 (7700; 10332)	3408 (2907; 3975)	0.01
CRP, mg/l	100.0 (20.6; 120.0)	18.0 (10.3; 74.5)	0.01
ESR, mm/h	46.0 (42.0; 63.0)	25.5 (12.0; 50.0)	0.01
Ferritin, mg/ml	1287.0 (326.0; 3509.0)	128.0 (51.0; 224.0)	0.0006
LDH, U/l	714.0 (635.0; 796.0)	464.0 (423.0; 513.0)	0.006
Total protein, g/dl	6.7 (6.2; 7.0)	7.2 (6.8; 7.8)	0.004
Albumin, g/dl	2.1 (1.8; 2.5)	3.1 (2.9; 3.3)	0.0003
Hepatomegaly	10 (90.9)	10 (38.5)	0.003
Splenomegaly	6 (54.6)	5 (19.2)	0.05
Lymphadenopathy	5 (45.5)	8 (30.8)	0.47
Coagulopathy	4 (36.3)	0 (0.0)	0.005*
Interstitial lung disease	5 (45.5)	1 (3.9)	0.005*
Heart involvement	6 (54.6)	4 (15,4)	0.04*
CNS dysfunction	6 (54.6)	0 (0.0)	0.0001*
MAS before TCZ (any time point)	7 (63.6)	5 (19.2)	0.018*
MAS immediately precede TCZ	5 (45.5)***	0 (0.0)	0.001*
**Outcomes during TCZ course**			
Corticosteroids discontinuation, n (%)	2/7 (28.6)	19/19 (100.0)	0.0001*
Corticosteroids discontinuation, days	167.0 (94.0; 237.0)	62.0 (34.0; 93.0)	0.005
Methotrexate discontinuation, due to remission of SoJIA, n (%)	1/8 (12.5)	8/24 (33.3)	0.39*
Cyclosporine A discontinuation, n (%)	3/7 (42.9)	8/13 (61.5)	0.64*
SoJIA relapses	5 (45.5)	2 (7.7)	0.016*
MAS during TCZ	5 (45.5)***	0 (0.0)	0.001*
Inactive disease	0 (0.0)	12/25** (48.0)	0.03*
TCZ-off remission	0 (0.0)	7/25** (28.0)	0.15*

*Fisher’s exact test, **the data analysis was performed on 25 patients (1 withdrew due to infusion reaction), ***the same patients whom TCZ was initiated during the MAS (MAS before TCZ and after)

**Figure 2:**
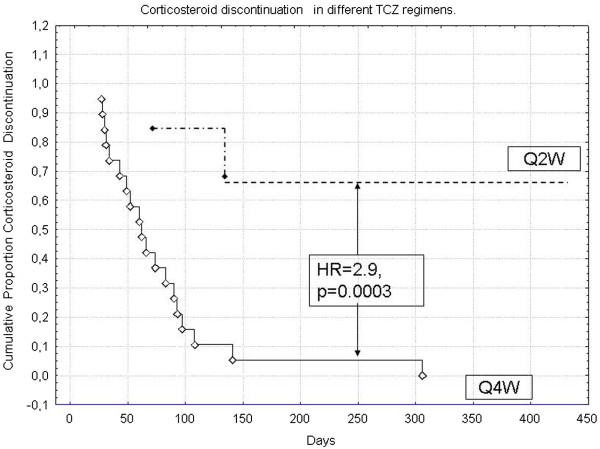
**Cumulative probability corticosteroid discontinuation in different TCZ regimens (Q2W vs. Q4W) between patients with Q4W and Q2W.**

The main interesting things were different outcomes in patients depending on activity. Patients from Q4W group were more susceptible to IL-6 blockade and had higher TCZ efficacy. All reached inactive disease and discontinued TCZ due to remission were from this group. No new cases of macrophage activation syndrome (MAS) were seen in the Q4W group as well as a lower frequency of organ involvement and relapses during TCZ treatment. Also, in the Q4W group, CS and MTX were discontinued more frequently and sooner compared to Q2W patients (HR = 2.9, p = 0.0003- Data in Table 2 and Figure 2). Only 1 child in the Q4W group developed a relapse after an increase of the time between TCZ infusions; We were able to re-start TCZ at q 4 weeks with the same efficacy as the first course.

No patients who required TCZ every 2 weeks experienced TCZ-free remission at the end of the study. Also, patients who required TCZ Q2W had MAS, hepatomegaly, splenomegaly, cardio-respiratory involvement, CNS dysfunction and coagulopathy more frequently than patients treated with TCZ Q4W. All cases of MAS relapses during TCZ therapy (only 1 relapse per patient) were in the Q2W group which likely reflects the severity of these patients.

We have found differences in the decrease of the % granulocytes at 1 week after the 1st TCZ infusion between the Q4W and the Q2Wgroups. Patients treated with Q4W TCZ decreased the granulocyte number by a factor of 2 while patients treated Q2W decreased the granulocytes by a factor of only 1.5. Possibly this difference in the decrease of the granulocytes may reflect the greater susceptibility to TCZ of the Q4W SoJIA patients. Patients from the Q4W group of patients also had less frequent infusion reactions.

As suspected, all patients who successfully discontinued TCZ were from the Q4W group and tolerated TCZ infusions every 4 weeks without relapse. One Q4W patient who had been off TCZ in remission for 1085 days experienced a relapse of the SoJIA and required a second course of TCZ with renewed efficacy. The clinical and laboratorial parameters of SJIA patients treated Q4W are in Table 3.

**Table 3:** **The cut-offs of clinical and laboratorial parameters of SJIA patients treated Q4W**

Parameter	OR (95%CI)	AUC** (95%CI)	p
Hemoglobin > 10.3 g/dl	32.0 (4.5; 227.2)	0.75 (0.57-0.88)	0.0001*
Granulocytes ≤ 9792 cells in 1 μl	24.8 (3.8; 160.0)	0.85 (0.68; 0.95)	0.0001*
WBC in 1 week ≤ 10.1*10^9^/l	6.9 (1.3; 37.2)	0.7 (0.49; 0.86)	0.027*
Granulocytes in 1 week ≤ 8142 cells in 1 μl	21.3 (2.9; 154.6)	0.82 (0.62-0.94)	0.001*
WBC in 2 weeks ≤ 7.5*10^9^/l	12.2 (2.1; 71.0)	0.66 (0.38; 0.88)	0.003*
Granulocytes in 2 weeks ≤ 3975 cells in 1 μl	28.0 (2.1; 379.3)	0.8 (0.52-0.96)	0.0015*
Platelets > 208 *10^9^/l	20.8 (2.0; 213.0)	0.64 (0.46; 0.8)	0.005*
CRP ≤ 82.2 mg/l	19.6 (3.3; 117.5)	0.77 (0.59-0.9)	0.001*
ESR ≤ 26 mm/h	28.4 (4.8; 168.3)	0.76 (0.58-0.89)	0.001*
Ferritin ≤ 605 mg/ml	89.9 (4.2; 1940.6)	0.86 (0.67-0.96)	0.0001*
LDH ≤ 571 U/l	42.0 (3.8; 469.1)	0.8 (0.58-0.94)	0.001*
Total protein > 7.2 g/dl	10.0 (1.1; 89.8)	0.75 (0.57-0.88)	0.027*
Albumin > 2.8 g/dl	46.0 (5.5; 382.7)	0.87 (0.7-0.96)	0.0001*
No splenomegaly	5.0 (1.1; 23.4)	-	0.05*
No hepatomegaly	31.8 (1.5; 659.6)	-	0.003
No coagulopathy	31.8 (1.5; 659.6)	-	0.005*
No ILD	20.8 (2.0; 213.0)	-	0.005*
No cardiac involvement	6.6 (1.3; 32.5)	-	0.04*
No CNS dysfunction	62.6 (3.1; 1282.9)	-	0.0001*
No kidney involvement	31.8 (1.5; 659.6)	-	0.005*
No MAS during TCZ	44.8 (2.2; 918.5)	-	0.01*

* Fisher’s exact test, ** AUC – area under the curve.

For selection of diagnostic criteria which can more accurately predict which patients might require Q2W TCZ rather than Q4W TCZ .

The clinical variables were:

1. Absence of hepatomegaly;
2. Coagulopathy;
3. CNS dysfunction; and
4. Interstitial lung disease;

The laboratory variables included:

1. Ferritin ≤ 605 μg/l,
2. LDH ≤ 571 U/l,
3. Albumin > 2.8 g/dl,
4. ESR ≤ 26 mm/h,
5. Granulocytes ≤9792 cells in 1 μl,
6. Hemoglobin > 10.3 g/dl,
7. Platelets > 208*109/l,
8. CRP ≤ 82.2 mg/l.

We evaluated separately the clinical and laboratory variables as well as combinations. We calculated sensitivity, specificity and OR for each combination. After this analysis, we found that only clinical variables and their combination (any 2 or moreclinical variables) had comparatively low OR - 44.8 (2.2 - 918.5), with low sensitivity level −0.45. Laboratory variables were more useful in the discrimination of Q4W then clinical characteristics. Any 4 or more laboratory criteria provided the highest specificity (1.0) and sensitivity (1.0) with highest OR – 1219.0 (22.8 – 65278.4) in the model. Any attempt to combine clinical variables with laboratory did not change sensitivity, specificity and mean of OR. Thus laboratory data in our patients appears to be more useful for predicting the patients with SoJIA who did well onQ4W rather than Q2W. Also we summarized the provisional clinical signs and laboratory parameters (risk factors) before 1st TCZ infusion which may increase the future probability of reaching remission off TCZ (Table 4): no fever before TCZ initiation, no MAS after start of TCZ, no lymphadenopathy, absence of coagulopathy and CNS dysfunction and low disease activity, allowed to use TCZ every 4 weeks.

**Table 4:** **Parameters, associated with achievement TCZ-off remission (Cox regression model)**

Risk factor	Hazard ratio	***P***-value ^§^
Fever before TCZ initiation: no (reference category: yes)	3.15	0.02
MAS after TCZ initiation: no (reference category: yes)	30.5	0.015
Lymphadenopathy before TCZ initiation: no (reference category: yes)	2.6	0.04
Coagulopathy before TCZ initiation: no (reference category: yes)	30.5	0.015
CNS dysfunction before TCZ initiation: no (reference category: yes)	3.8	0.04
Low diseases activity: TCZ Q2W (reference category: Q4W)	0.22	0.004

Event of interest: achievement TCZ-off remission. Complete data were available for 33 patients.

^§^P values refer to the Wald’s chi^2^-test.

## Discussion

TCZ (an anti-IL-6) is one of the new biologic drugs that has been approved in some countries for treatment of SoJIA patients as young as 2 years. Another effective class of biologics, which shows a similar efficacy, is the Il-1 blockers.

Unfortunately, in some countries such as Russia, the IL-1 blockers are still unavailable in 2014 and TCZ is the only biologic option in treatment of SoJIA. According to the results of two published randomized control trials, treatment with TCZ can achieve inactive disease in more than 1/3 patients with SoJIA [4–6].

Improvement in SoJIA course or achievement status of inactive disease allows to the tapering and often discontinuation of CS and DMARDS. It is known that patients with SoJIA are variable in disease course and severity. The SoJIA disease courses range from a benign monocyclic course with mild arthritis to a relapsing and persistent course with severe articular and extra-articular damage [20]. These different disease courses are the basis for choosing different treatment strategies. Themilder patients usually require small doses of NSAIDs and CS while severe patients need higher doses of CS and deep and prolonged immunosuppression [21]. In the study of M. Gattorno et al., it was shown that there appeared to be 2 subsets of SoJIA patients with different response on Il-1 blockade. One subset of patients, who had more systemic features and had fewer active joints, appeared to have a better response to the Il-1 medication [22].

Our idea was similar. Children with SoJIA may well have different treatment responses to TCZ, and we set out to find the group of patients which might be more responsiveto TCZ treatment.

The dosage and regimen of TCZ appears to depend on the disease itself and the disease pathogenesis. Children with Castleman’s disease require a TCZ dosage of 8 mg/kg weekly because in this systemic diseaselymphocytes and macrophages of the affected lymph nodes continuously produce IL-6 [23, 24].

It is useful to compare SoJIA to adult rheumatoid arthritis (RA). In children with SoJIA, a very impressive systemic inflammation occurs, presumably provided by cells of innate immunity of bone marrow origin. In adult RA, IL-6 is produced by synoviocytes in the inflamed joints. The IL-6 is part of a group of inflammatory cytokines produced in RA such as TNF-α, IL-1β, and other cytokines. RA appears to be more a restricted and not as a systemic inflammatory disease targeting more the joints than other organs, at least compared to SoJIA. In RA, TCZ is able to be infused at an every 4-week interval [25].

The extent of immunological activation appears to be more potent in SoJIA compared to RA, which supports the rationale to use TCZ Q2W in very active, systemically ill SoJIA patients. Indeed, as the pathophysiological background of each disease appears to be very different, the justification for different infusion intervals is clear.

The previous data about the pharmacokinetics of TCZ in SoJIA have shown the efficacy after a single TCZ administration in doses ranging from 2 to 12 mg/kg, with continued effectiveness up to 8 weeks after a dose [11]. Also there is data supporting the effectiveness of even lower doses of TCZ: 8 mg/kg instead of 12 mg/kg and 4 mg/kg instead of 8 mg/kg. It has also been shown that the time between infusions could be increased up to 3 weeks the without TCZ failure [26]. One of our aims was to distinguish the subgroup of patients with a milder SoJIA course and a higher responsiveness to TCZ for whom TCZ treatment every 4 weeks will be quite effective.

For the lower disease activity group, or mild SoJIA, TCZ infused every 4 weeks may well be sufficient. These patients will be also treated with the conventional protocol such as the low-dose corticosteroid management, or they might have been a patient with a monocyclic SoJIA course type.

One weakness of our study is that identification of the low disease activity SoJIA group is problematic as patients often receive concomitant anti-rheumatic treatment, such as CS, MTX, and CsA, before entering into the tocilizumab treatment. Whether the lower disease activity group really exists, or their disease activity was lowered by the previous treatment,or both, is hard to distinguish and may influence our judgment on the efficacy of tocilizumab in each patient. Yet in our trial there were impressive differences that support the reality of a lower disease activity group. In the patient group with low disease activity who were treated every 4 weeks, we could decrease CS more frequently and faster, had less frequency of relapses, had better response rates, more quickly achieved a status of inactive disease and TCZ-free remission. We believe that these treatment responses and outcomes support the likelihood of a low disease activity group in our SoJIA patients at our center.

The elongation of intervals between infusions without failure of efficacy has a lot of benefits. We have to be aware of the cost-benefit ratio. Also, less frequent infusions improves the patient’s and family’s quality of life with less frequent outpatient infusions or hospital infusions.

There are other limitations of our study. It is a non-blinded retrospective cohort study with all the limitations of such a study. In any single center’s experience with TCZ, there is a problem with a smaller sample size which may bias our results. The observation period after the TCZ infusions is also relatively short. These criteria are useful for our patients and it remains to be seen if it proves useful in larger series of patients and at other centers.

## Conclusions

In our study we have shown a comparative efficacy of different TCZ protocols based on clinical heterogeneity of SoJIA patients. We offer a set of provisional criteria that may help differentiate patients with favorable and unfavorable disease courses and allow TCZ treatment every 4 weeks for the patients with the more favorable prognosis instead of the more aggressive every 2 week regimen. Our criteria may help increase the probability of a child with SoJIA reaching remission off of TCZ without the expense and potential side-effects of using TCZ every 2 weeks.

### Ethics

Written consent was obtained according to the declaration of Helsinki. Approval of the protocol of this trial was approved by local Ethic Committee of Saint-Petersburg State Pediatric Medical University.

## Abbreviations

ACRPedi70: American College of Rheumatology Pediatric criteria
CRP: C-reactive protein
CS: Corticosteroids
CsA: Cyclosporine A
DMARDs: Disease-modifying antirheumatic drugs
ESR: Erythrocyte sedimentation rate
Hb: Hemoglobin
HR: hazard ratio
Il-1: interleukine-1
Il-6: Interleukine-6
ILAR: International League Against Rheumatism
IQR: Interquartile ranges
LDH: Lactate dehydrogenize
MAS: Macrophage activation syndrome
Me: Median
MTX: Methotrexate
OR: Odds ratio
PLT: Platelets
Q2W: Every 2 weeks
Q4W: Every 4 weeks
RA: Rheumatoid arthritis
SoJIA: Systemic juvenile idiopathic arthritis
TCZ: Tocilizumab
WBC: White blood cells.
